# Glucocorticoids control systemic inflammatory response by regulation of energy metabolism and cytokine expression

**DOI:** 10.1186/cc11787

**Published:** 2012-11-14

**Authors:** A Kleyman, S Hübner, M Bauer, JP Tuckermann

**Affiliations:** 1 Center for Sepsis Control and Care, Jena University Hospital, Jena, Germany; 2 Leibniz Institute for Age Research, Jena, Germany; 3 University of Ulm, Institute for General Zoology and Endocrinology, Ulm, Germany

## Background

Endogenous glucocorticoids (GCs) are essential for survival in systemic inflammatory response (SIRS). So far, their effect has been attributed exclusively to anti-inflammatory activities of GCs in leukocytes. GCs act through glucocorticoid receptor (GR), a ubiquitously expressed nuclear receptor. GR binds as a homodimer to the glucocorticoid responsive element in the promoter of target genes and regulates their transcription. Another mode of GR action is dimerization independent; monomeric GR interacts with other transcriptional factors such as AP1, NF-κB, IRF3, modulates their activity and in this way regulates the expression of target genes. Our study explored which cells mediate the beneficial effects of GCs and identified GR target genes important for survival in systemic inflammatory response.

## Methods

Knock-in mice carrying a dimerization-deficient GR (GRdim mice), leptin-deficient mice (ob/ob mice) and the respective wild-type littermates were subjected to a LPS-induced shock model.

## Results

After high-dose LPS (10 mg/kg) wild-type mice decreased oxygen consumption and developed mild temporal hypothermia; however, in 6 to 12 hours they discontinued to decrease energy metabolism and recover during 24 to 48 hours. Recovery of energy metabolism in wild-type mice was accompanied by upregulation of leptin expression in white adipose tissue and an increase of leptin serum level as well as upregulation of expression of the leptin target gene (proopiomelanocortin (POMC)) in the hypothalamus. After LPS injection, mice with a dimerization-deficient GR (GRdim mice) had normal TNFα and elevated IL-1β serum level compared with wild-type littermates. GRdim mice developed a strong repression of oxygen consumption, severe hypothermia and died during 48 hours after LPS administration. Continued repression of energy metabolism in GRdim mice was associated with the inability to increase leptin serum level and a misregulation of leptin signaling in hypothalamus after LPS injection. Leptin-deficient ob/ob mice demonstrate phenotype similar to GRdim mice: a strong suppression of energy metabolism, low oxygen consumption, severe hypothermia, and increased lethality in systemic inflammation. Importantly, treatment with IL-1 receptor antagonist (IL-1RA) alone does not rescue GRdim mice; however, simultaneous treatment with IL-1RA and leptin significantly increase energy metabolism and rescue survival of GRdim mice in LPS-induced SIRS.

## Conclusion

We show that endogenous GCs fulfill their protective function during systemic inflammation by different mechanisms: repression of cytokine expression and regulation of energy metabolism via upregulation of leptin expression.

